# Patterns of failure in gastric carcinoma after D2 gastrectomy and chemoradiotherapy: a radiation oncologist's view

**DOI:** 10.1038/sj.bjc.6601896

**Published:** 2004-05-25

**Authors:** D H Lim, D Y Kim, M K Kang, Y I Kim, W K Kang, C K Park, S Kim, J H Noh, J W Joh, S H Choi, T S Sohn, J S Heo, C H Park, J O Park, J E Lee, Y J Park, H R Nam, W Park, Y C Ahn, S J Huh

**Affiliations:** 1 Department of Radiation Oncology, Samsung Medical Center, Sungkyunkwan University School of Medicine, 50 Ilwon-dong, Kangnam-gu, Seoul, 135-710, Korea; 2 Department of Surgery, Samsung Medical Center, Sungkyunkwan University School of Medicine, 50 Ilwon-dong, Kangnam-gu, Seoul, 135-710, Korea; 3 Division of Hematology-Oncology, Department of Medicine, Samsung Medical Center, Sungkyunkwan University School of Medicine, 50 Ilwon-dong, Kangnam-gu, Seoul, 135-710, Korea; 4 Department of Pathology, Samsung Medical Center, Sungkyunkwan University School of Medicine, 50 Ilwon-dong, Kangnam-gu, Seoul, 135-710, Korea

**Keywords:** gastric adenocarcinoma, chemoradiotherapy, pattern of failure

## Abstract

The risk of locoregional recurrence in resected gastric adenocarcinoma is high, but the benefit of adjuvant treatment remains controversial. In particular, after extended lymph node dissection, the role of radiotherapy is questionable. Since 1995, we started a clinical protocol of adjuvant chemoradiotherapy after D2 gastrectomy and analysed the patterns of failure for 291 patients. Adjuvant chemotherapy consisted of five cycles of fluorouracil and leucovorin, and concurrent radiotherapy was given with 4500 cGy from the second cycle of chemotherapy. With a median follow-up of 48 months, 114 patients (39%) showed any type of failure, and the local and regional failures were seen in 7% (20 out of 291) and 12% (35 out of 291), respectively. When the recurrent site was analysed with respect to the radiation field, in-field recurrence was 16% and represented 35% of all recurrences. Our results suggest that adjuvant chemoradiotherapy has a potential effect on reducing locoregional recurrence. Moreover, low locoregional recurrence rates could give a clue as to which subset of patients could be helped by radiotherapy after D2 gastrectomy. However, in order to draw a conclusion on the role of adjuvant radiotherapy, a randomised study is needed.

Gastric cancer is the most common cancer in Korea (Shin *et al*, 2002). Complete tumour removal with sufficient resection margin plus extended lymph node dissection is considered to be the most important factor in terms of reduced locoregional recurrence and improved survival. Although the incidence of early gastric cancer is increasing, most patients still present with an advanced stage, and, even after complete resection, 38–94% of patients developed locoregional recurrence (McNeer *et al*, 1951; Gunderson and Sosin, 1982; Wisbeck *et al*, 1986; Landry *et al*, 1990), which translates into 33–69% of all recurrences (Gunderson and Sosin, 1982; Landry *et al*, 1990; Maehara *et al*, 2000; Yoo *et al*, 2000). However, high recurrence rates may be reduced by adjuvant treatment, so postoperative adjuvant radiotherapy and/or chemotherapy is generally performed.

There were several studies (GITSG, 1982; Moertel *et al*, 1984; Allum *et al*, 1989; Regine and Mohiuddin, 1992) showing positive effects of radiotherapy and/or chemotherapy in patients with locally advanced or unresectable gastric adenocarcinoma, but the benefit of adjuvant treatment after curative resection remains controversial. Intergroup trial (MacDonald *et al*, 2001) showed that adjuvant chemoradiotherapy reduced recurrences and increased survival of patients with gastric adenocarcinoma. However, after extended lymph node dissection with R0 gastrectomy, there are no data regarding whether adjuvant radiotherapy could reduce locoregional recurrence and increase survival.

The purpose of this study was to analyse the patterns of failure in patients with gastric adenocarcinoma after gastrectomy with D2 lymph node dissection plus postoperative chemoradiotherapy, and to evaluate any effect of adjuvant radiotherapy on locoregional recurrence reduction and survival improvement.

## MATERIALS AND METHODS

### Patients

From 1995 to 1999, a total of 322 patients with curatively resected gastric adenocarcinoma received adjuvant chemoradiotherapy at the Samsung Medical Center. The eligibility criteria and the postoperative adjuvant treatment used were the same as those used for the SWOG-9008 (INT-0116) trial (MacDonald *et al*, 2001). All patients had R0 gastrectomy and the extent of lymph node dissection was more than D2 dissection. At 3–7 weeks after surgery, chemotherapy with fluorouracil 400 mg m^−2^ day^−1^ and leucovorin 20 mg m^−2^ day^−1^ was administered for 5 days, and this was followed by chemoradiotherapy beginning 4 weeks after the start of the first cycle of chemotherapy. The chemoradiotherapy consisted of 4500 cGy of radiation over 5 weeks, with fluorouracil and leucovorin on the first 4 and last 3 days of radiotherapy. At 4 weeks after the completion of radiotherapy, two 5-day cycles of chemotherapy with fluorouracil plus leucovorin were given 4 weeks apart. Initially, the pathologic stage was classified according to the 1992 staging criteria of the American Joint Commission on Cancer, but this was refined in accord with the 1997 AJCC staging criteria. Informed consent was obtained from each individual before enrolment and the study protocol was reviewed and approved by the Samsung Medical Center Institutional Review Board.

### Radiotherapy plan

**Figure 1:**
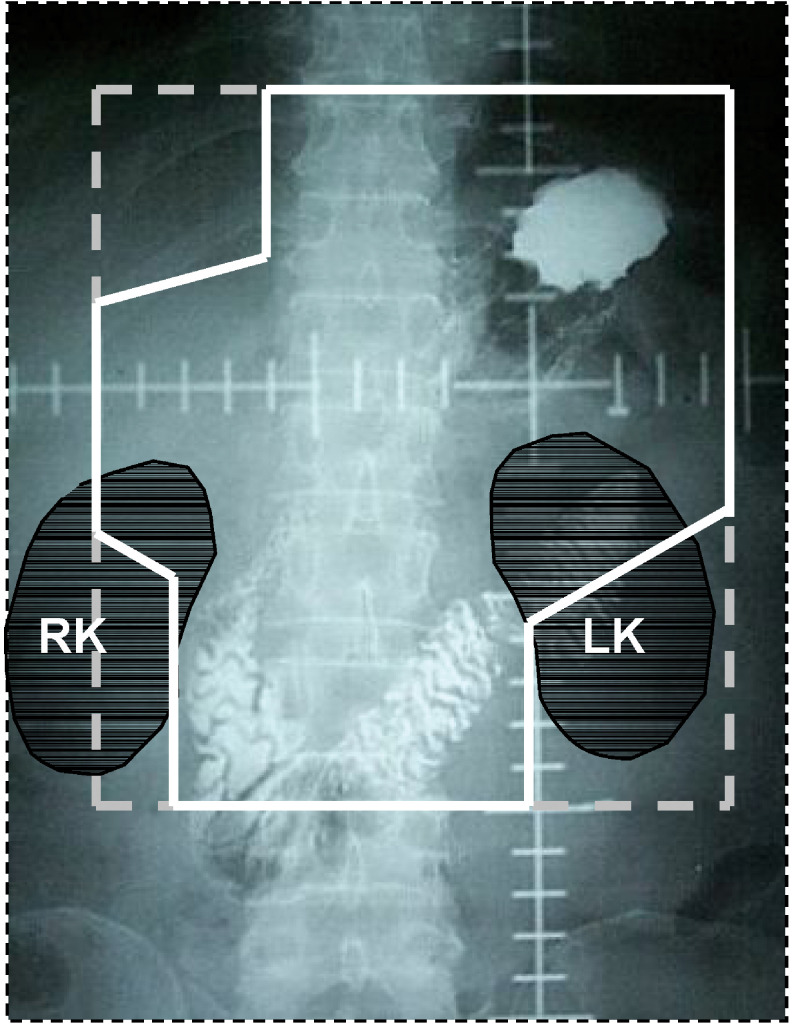
Radiation field of a patient with subtotal gastrectomy and Billroth I anastomosis. The white solid line includes the tumour bed, anastomosis site, remnant stomach and regional lymph nodes. RK=right kidney; LK=left kidney.

Radiation was targeted to the tumour bed, anastomosis site, duodenal stump, remnant stomach and regional lymph nodes (Figure 1

). Other than for T4 lesions, the tumour bed was not the radiotherapy target, because there was no microscopic residuum after R0 gastrectomy. For the radiation of the anastomosis site and duodenal stump, more than 2 cm beyond the proximal and distal resection margins was included within the radiation field. In patients with subtotal gastrectomy, the remnant stomach was included in the radiation field, but, if the volume of the irradiated left kidney exceeded more than half of the whole left kidney volume or if a patient could not tolerate the acute gastrointestinal complications caused by the large radiation field, the field was modified, and some of the remnant stomach was excluded. To delineate regional lymph node areas, we used the definition of the Japanese Research Committee for Gastric Cancer (Kajitani, 1981). Although the irradiated node areas were modified according to the primary tumour location, the radiation field usually included more than group 2 lymph nodes. Anterior–posterior parallel opposing fields were used and the radiation dose was 4500 cGy, with 180 cGy daily fractions over 5 weeks. If the proximal or distal resection margin was less than 2 cm, a boost radiotherapy of 1000 cGy was delivered in daily fractions of 200 cGy.

### Follow-up and definition of recurrence

After the completion of planned adjuvant treatment, regular follow-up was carried out with physical examination, complete blood count, liver function test, chest radiography, abdominopelvic computed tomography and gastroscopy. The follow-up intervals were 3 months for the first year, 6 months for the next 2 years and yearly thereafter. During the follow-up period, any suspected recurrence was confirmed pathologically, if possible, and the first recurrence site was used to determine local recurrence, regional recurrence, or distant metastasis. Local recurrence was defined as recurrence at the anastomosis site, duodenal stump, tumour bed, or remnant stomach. Regional recurrence was defined as recurrence at the regional lymph nodes within the radiation field. Distant metastasis was defined as lymph node recurrence outside the radiation field, peritoneal seeding, liver metastasis, or metastasis of other extra-abdominal sites. If two or more failure sites developed at the same time, they were counted separately.

### Statistical methods

Relapse-free survival was defined as the time from surgery to the first recurrence and overall and disease-free survival was estimated by the Kaplan–Meier method. To determine relationships between the patterns of recurrence and clinicopathological factors, the *χ*^2^ test was used. Independent risk factors that influenced recurrence were analysed for by logistic regression analysis.

## RESULTS

### Patients' characteristics

Of the 322 patients enrolled on the adjuvant chemoradiotherapy protocol, 31 (10%) did not receive radiotherapy because of a poor performance status, refusal of further treatment or disease progression after the first cycle of chemotherapy. Therefore, 291 patients were analysed for survival and patterns of failure. The median follow-up period for all patients was 48 (range 5–84) months, and that of living patients was 60 (range 37–84) months.

The characteristics of the patients and their surgical results are described in Tables 1

**Table 1:** Characteristics of the patients (*n*=291)

**Characteristics**	**No. of patients (%)**
*Sex*	
Male	190 (65)
Female	101 (35)
	
*Age*	
⩽60	210 (72)
>60	81 (28)
	
*ECOG performance score*	
0–1	279 (96)
2–3	12 (4)
	
*Location of tumour*	
Distal 1/3	145 (50)
Middle 1/3	120 (41)
Proximal 1/3	23 (8)
Whole stomach	3 (1)
	
*Type of operation*	
Total gastrectomy	116 (40)
Subtotal gastrectomy	175 (60)

and 2

**Table 2:** Postoperative results of patients (*n*=291)

**Characteristics**	**No. of patients (%)**
*Histology*	
WHO classification	
Adenocarcinoma^a^	215 (74)
Mucinous	15 (5)
Signet ring cell	59 (20)
Undifferentiated	2 (1)
	
*Grade*	
W/D	4 (1)
M/D	82 (28)
P/D	205 (70)
	
*Lauren type*	
Intestinal	92 (32)
Diffuse	190 (65)
Mixed	9 (3)
	
*Stage*^b^	
T stage	
T1	25 (9)
T2	116 (40)
T3	143 (49)
T4	7 (2)
N stage	
N0	28 (10)
N1	135 (46)
N2	83 (29)
N3	45 (16)
AJCC	
IB	35 (12)
II	72 (25)
IIIA	91 (31)
IIIB	46 (16)
IV (M0)	47 (16)

W/D=well differentiated; M/D=moderately differentiated; P/D=poorly differentiated.

a Papillary and tubular adenocarcinoma.

b 1997 AJCC staging criteria.

. The numbers of dissected lymph node were 10–114 (median, 35), and in 236 patients (81%) more than 25 lymph nodes were dissected. Lymph node metastasis developed in 90% of the patients (263 out of 291 patients). Eight patients (3%) did not complete radiation therapy because of gastrointestinal complications, such as nausea and diarrhoea in four patients, three refused radiotherapy for personal reasons and one patient developed distant metastasis during radiotherapy.

### Toxicity

**Figure 2:**
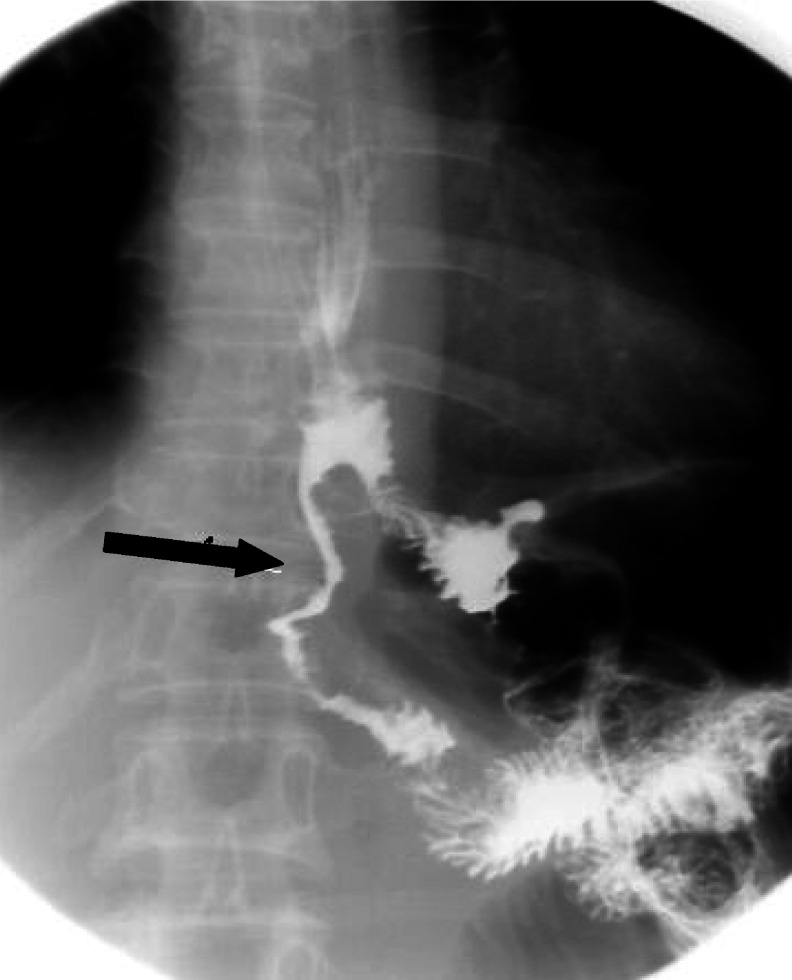
Upper gastrointestinal radiograph of a patient who developed intestinal radiation fibrosis. The black arrow indicates segmental narrowing of the jejunal loop below the anastomosis site.

Toxicities were graded as 1–4 based on the Southwest Oncology Group Toxicity Criteria. In terms of gastrointestinal complications, nausea was the most common toxicity and 36 patients (12%) experienced nausea graded as 3 or higher. Diarrhoea graded as 3 or higher occurred in 30 patients (10%). Four patients did not complete radiotherapy because of these gastrointestinal complications. During the follow-up period, intestinal fibrosis associated with radiotherapy developed in one patient (Figure 2

) and intestinal obstructions not due to tumour recurrence occurred in 20 patients – 15 patients as grade 3 and five patients as grade 4. The resection and re-anastomosis or adhesiolysis was performed in five patients (2%) with intestinal obstruction grade 4. In three of these five patients, the bowel obstructive sites were outside the radiation field. The most common hematologic complication was neutropenia, and 90 patients (31%) had neutropenia graded as 3 or higher. Thrombocytopenia graded as 3 or higher occurred in 15 patients (5%). No toxicity-related death occurred during follow-up period.

### Survival and relapse

**Figure 3:**
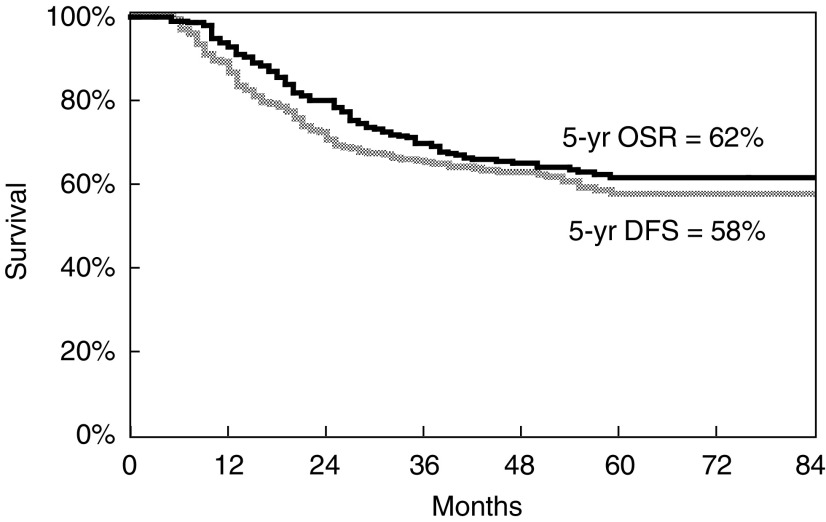
Overall survival (5-year) and disease-free survival of all patients (*n*=291).

The 5-year overall survival and disease-free survival rates were 62 and 58% (Figure 3

). Figure 4

**Figure 4:**
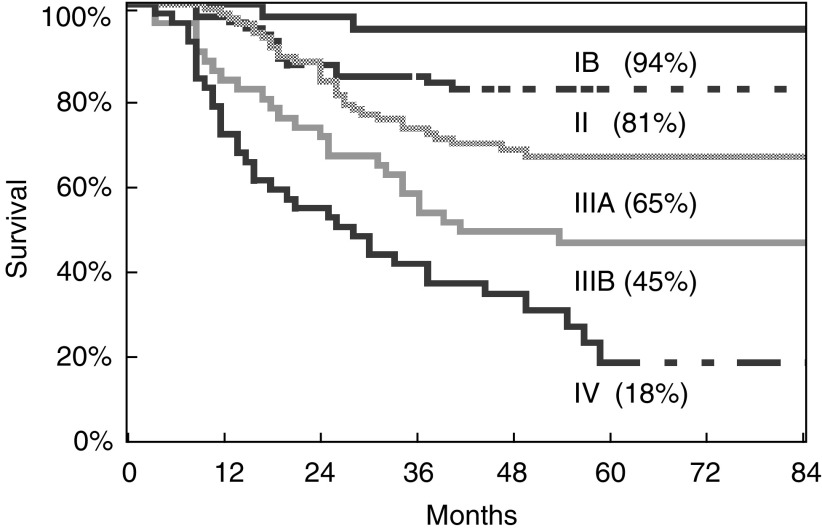
Overall survival (5-year) according to AJCC stage.

shows the 5-year survival rates according to AJCC stage.

**Figure 5:**
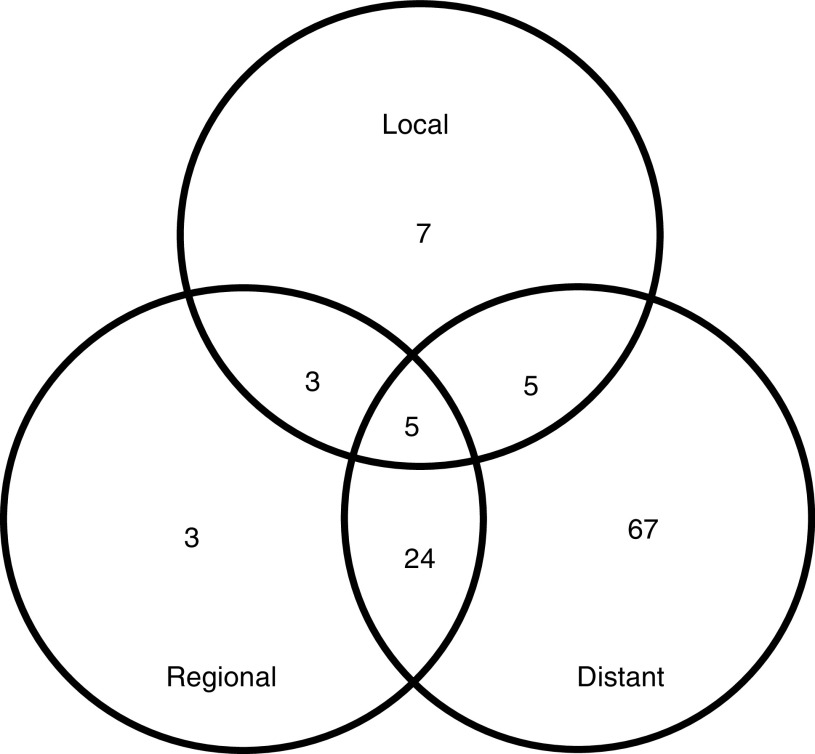
Patterns of failure in 114 patients: local recurrence in 20 patients, regional recurrence in 35 patients, distant metastasis in 101 patients.

During the follow-up period, 114 patients (39%) relapsed at 156 sites and the median time to recurrence from surgery was 16 months (ranged 3–59 months). Of these patients, 77 patients had one recurrence pattern – local recurrence in seven patients, regional recurrence in three patients and distant metastasis in 67 patients, and 37 patients had recurrences at multiple sites (Figure 5

). Therefore, local recurrence, regional recurrence and distant metastasis occurred in 20 patients (7% of all patients), 35 patients (12% of all patients) and 101 patients (35% of all patients), respectively. Locoregional recurrence, defined as recurrence within the radiation field, developed in 16% (47 out of 291) and represented 35% of all recurrences. In all, 65% of recurrences developed outside the radiation field and the most common metastatic site was peritoneal seeding. Table 3

**Table 3:** The recurrent sites (156 sites) of 114 patients

**Recurrent site**	**No. of patients (%)**
Local failure	20 (7)
Anastomosis site	16
Remnant stomach	4
Duodenal stump	2
Regional failure	35 (12)
Distant metastasis	101 (35)
Peritoneal seeding	81
Liver metastasis	23
Pleura+lung parenchyma	11
Bone metastasis	7
Virchow’s node	6
Brain metastasis	2

shows the specific recurrence sites.

### Prognostic factors for recurrence

To evaluate the prognostic factors of recurrence, variable clinicopathological factors – sex, age, type of operation, location of tumour, histological type (WHO classification, grade, Lauren type), invasion depth of tumour, number of involved lymph node and TNM stage – were analysed. The type of operation, the location of tumour and its stage were prognostic factors for total recurrence, but sex, age and histological type were unrelated to recurrence (Table 4

**Table 4:** The patterns of failure according to clinicopathological factors

	**Total recurrence**	**Local recurrence**	**Regional recurrence**	**Distant metastasis**
*Type of operation*				
TG (*n*=116)	61/116 (53%)	9/116 (8%)	19/116 (16%)	53/116 (46%)
STG (*n*=175)	53/175 (30%)	11/175 (6%)	16/175 (9%)	48/175 (27%)
*P*	**0.0001**	ns	ns	**0.001**
				
*Location of tumour*				
Distal (*n*=150)	57/150 (38%)	10/150 (7%)	23/150 (15%)	52/150 (35%)
Middle (*n*=123)	43/123 (35%)	8/123 (7%)	7/123 (6%)	38/123 (31%)
Proximal (*n*=15)	11/15 (73%)	2/15 (13%)	4/15 (27%)	8/15 (53%)
Whole (*n*=3)	3/3 (100%)	0/3 (0%)	1/3 (33%)	3/3 (100%)
*P*	**0.049**	ns	ns	ns
				
*T stage*				
T1 (*n*=25)	1/25 (4%)	1/25 (4%)	1/25 (4%)	1/25 (4%)
T2 (*n*=116)	30/116 (26%)	6/116 (5%)	11/116 (10%)	25/116 (22%)
T3 (*n*=143)	77/143 (54%)	10/143 (7%)	21/143 (15%)	70/143 (49%)
T4 (*n*=7)	6/7 (86%)	3/7 (43%)	2/7 (29%)	5/7 (71%)
*P*	**<0.0001**	**0.045**	**0.033**	**<0.0001**
				
*N stage*				
N0 (*n*=28)	6/28 (21%)	2/28 (7%)	1/28 (4%)	5/28 (18%)
N1 (*n*=135)	33/135 (24%)	6/135 (4%)	9/135 (7%)	30/135 (22%)
N2 (*n*=83)	40/83 (48%)	5/83 (6%)	12/83 (15%)	34/83 (41%)
N3 (*n*=45)	35/45 (78%)	7/45 (16%)	13/45 (29%)	32/45 (71%)
*P*	**<0.0001**	**0.06**	**<0.0001**	**<0.0001**
				
*TNM stage*				
IB (*n*=35)	3/35 (9%)	2/35 (6%)	1/35 (3%)	2/35 (6%)
II (*n*=72)	11/72 (15%)	2/72 (3%)	2/72 (3%)	11/72 (15%)
IIIA (*n*=91)	37/91 (41%)	5/91 (6%)	13/91 (14%)	32/91 (35%)
IIIB (*n*=46)	26/46 (57%)	4/46 (9%)	5/46 (11%)	22/46 (48%)
IV (*n*=47)	37/47 (79%)	7/47 (15%)	14/47 (30%)	34/47 (72%)
*P*	**<0.0001**	**0.02**	**<0.0001**	**<0.0001**

TG=total gastrectomy; STG=subtotal gastrectomy.

). The TNM stage and the invasion depth of a tumour (T stage) were found to correlate with all types of recurrent patterns. The number of involved lymph nodes (N stage) did not affect local recurrence, but did affect regional recurrence and distant metastasis.

Logistic regression analysis showed that the independent risk factors for total recurrence were tumour invasion to more than the serosa, total gastrectomy, and lymph node metastasis. When each recurrence site was separately analysed, no statistically significant risk factor for local recurrence was identified. For regional recurrence, total gastrectomy was the only risk factor, and for distant metastasis tumour invasion to more than the serosa, lymph node metastasis and total gastrectomy were risk factors.

## DISCUSSION

To determine the role of postoperative adjuvant radiotherapy in resected gastric adenocarcinoma, a comparative analysis of failure patterns after curative resection with and without adjuvant treatment is needed. The patterns of failure after curative resection in gastric cancer have been reported on many occasions (McNeer *et al*, 1951; Thomson and Robins, 1952; Wangensteen *et al*, 1954; Gunderson and Sosin, 1982; Wisbeck *et al*, 1986; Landry *et al*, 1990; Yoo *et al*, 2000), and these studies show high levels of locoregional recurrences after curative resection. However, we should be cautious about accepting the results of these studies.

Many studies that reported high locoregional recurrences have some drawbacks in terms of data comparisons. First, they had different viewpoints of recurrence. For example, the results of autopsy studies provide useful information about the recurrence, but they reveal only the end patterns of failure after surgery (McNeer *et al*, 1951; Thomson and Robins, 1952; Wisbeck *et al*, 1986). Reoperation data are highly selective, with a focus on high-risk patients (Wangensteen *et al*, 1954; Gunderson and Sosin, 1982), and clinical studies contain relatively little information on the exact incidence of recurrence, though they are useful for assessing which subsets of patients may benefit from adjuvant therapy (Papachristou and Fortner, 1981; Landry *et al*, 1990; Maehara *et al*, 2000; Yoo *et al*, 2000). Second, the definition of failure patterns differed, in particular, the definition of regional recurrence. In the present study, we defined the regional recurrence as recurrence at lymph nodes within the radiation field, and the radiation field usually included more than group 2 lymph nodes. The lymph node recurrence outside the radiation field was defined as distant metastasis. The definition of our study accords with the concept that metastasis to group 3 lymph nodes could be considered as distant metastasis. Third, the surgical extent differed, especially with respect to the extent of lymph node dissection. Though the effect of extended lymph node dissection remains controversial between randomised studies (Dent *et al*, 1988; Bonenkamp *et al*, 1999; Cuschieri *et al*, 1999) and retrospective studies (Kodama *et al*, 1981; Pacelli *et al*, 1993; Siewart *et al*, 1998), the extent of lymph node dissection may affect regional recurrence, which causes confusion when we attempt to determine the role of postoperative radiotherapy. Therefore, from studies on patterns of failure after curative resection, we can obtain useful but limited information about locoregional recurrence and the determination of the radiotherapy target. Thus, in order to determine the role of adjuvant radiotherapy in resected gastric adenocarcinoma, a well-organised randomised study is needed. In this respect, the results of the intergroup trial were encouraging.

MacDonald *et al* reported the results of a randomised trial. They compared chemoradiotherapy after surgery with surgery alone in adenocarcinoma of the stomach or gastroesophageal junction. This was the only randomised study to compare adjuvant chemoradiotherapy with surgery alone. The study showed that overall survival and relapse-free survival were superior in the adjuvant chemoradiotherapy group, whereas the relapse rate was higher in the surgery alone group. They concluded that postoperative chemoradiotherapy should be considered for all patients with a high risk of recurrence. This randomised study showed that adjuvant chemoradiotherapy has a significant role in reducing recurrence and increasing survival, and suggests that it could become a standard treatment modality in resected gastric adenocarcinoma. However, this result does not apply to all cases of resected gastric adenocarcinoma. As only 10% of the patients of intergroup trial had undergone D2 lymph node dissection, we have limited information as to whether another locoregional modality plays a role after a primary locoregional modality in gastric adenocarcinoma with D2 gastrectomy. Therefore, we believe that it is unreasonable to conclude that postoperative chemoradiotherapy should be administered to patients with gastrectomy plus D2 lymph node dissection. Another considerable point concerns the identity of the subset of patients at high risk of locoregional recurrence. In many retrospective studies (Kodama *et al*, 1981; Maruyama *et al*, 1989; Sasako *et al*, 1995), the invasion depth of the primary tumour was found to influence the extent of lymph node metastasis, so there is a possibility that not all stage patients would benefit from adjuvant radiotherapy after extended lymph node dissection.

Even after complete resection, 38–94% of patients developed locoregional recurrence (McNeer *et al*, 1951; Gunderson and Sosin, 1982; Wisbeck *et al*, 1986; Landry *et al*, 1990) and this accounted for 33–69% of all recurrences (Gunderson and Sosin, 1982; Landry *et al*, 1990; Maehara *et al*, 2000; Yoo *et al*, 2000). These wide recurrence rates are caused by the different criteria used for recurrence, and the recurrence rates of clinical series are lower than those of reoperation or autopsy studies. In our study, patterns of failure were analysed according to the radiation field. Locoregional recurrence developed in 16% of all treated patients, and local and regional recurrences comprised 13 and 22% of all recurrences. Some clinical series have reported similar results. According to Yoo *et al*, local and regional recurrences were 18 and 13% of all recurrences, and Maehara *et al* reported a local recurrence of 22% and a regional recurrence of 12%, as percentages of all recurrence. These two studies are worthy of note because the extent of surgery was D2 gastrectomy, as in the present study. Though their studies were clinically based, the relatively low rates of locoregional or regional recurrence observed may be related to extended lymph node dissection. However, analysed patients were selected from all recurrent patients, and no statement was made concerning locoregional recurrence among resected patients. Therefore, we can say that locoregional recurrence accounts for about one-third of total recurrences in some clinical series, but it is difficult to conclude that similar locoregional recurrence rates mean no need for adjuvant radiotherapy. In our study, local recurrence was 7% and regional recurrence was 12% for all treated patients.

In most studies that have analysed the patterns of failure, the most common local failure site was the anastomosis or stump. Landry *et al* reported that the recurrence rate of anastomosis was 25% in all patients. In subtotal gastrectomy patients, Papachristou and Fortner reported that the remnant stomach was the highest local recurrence site, with a 22% recurrence rate. According to our data, recurrences at the anastomosis site and at the remnant stomach were only 5% (16 out of 291) and 2% (four out of 175), respectively. These low local recurrence rates might be due to surgical factors or adjuvant treatment.

Many risk factors for locoregional recurrence have been reported and the degree of gastric wall penetration has been related to locoregional recurrence (Gunderson and Sosin, 1982; Landry *et al*, 1990; Maehara *et al*, 2000; Yoo *et al*, 2000). This was also observed in the present study and the invasion depth of tumour correlated to all types of recurrences. The relation between invasion depth and recurrence results from the abundant lymphatic and vascular vessels in the gastric wall and tumour extension by these routes (Lehnert *et al*, 1985). Otherwise, the number of involved lymph nodes was found to be correlated to regional recurrence and distant metastasis, but not to local recurrence. Multivariate analysis showed that tumour invasion more than the serosa or lymph node metastasis are risk factors for total recurrence. That is, patients with T3, T4 and/or lymph node metastasis could be candidates for adjuvant radiotherapy or chemoradiotherapy.

The purpose of this study was to analyse the patterns of failure after postoperative adjuvant chemoradiotherapy after gastrectomy with D2 lymph node dissection, and determine whether adjuvant radiotherapy reduces locoregional recurrence and increases survival. Our study is based on clinical and radiological examinations and is not a randomised study. Therefore, there is a possibility of underestimating the recurrence pattern, and it is difficult to conclude that postoperative chemoradiotherapy could affect the recurrence pattern or survival after curative resection in gastric adenocarcinoma. However, this is the first study to analyse the patterns of failure after chemoradiotherapy in patients with gastrectomy and D2 lymph node dissection. Locoregional recurrence developed in 16% of all patients, and, excepting patients with T4 or N3, less than 15% of the local or regional recurrences developed (Table 4). We do not know whether these recurrence results are due to surgical extent, which included extended lymph node dissection or adjuvant chemoradiotherapy, or both. However, the results of low locoregional recurrence rates could give a clue as to which subset of patients could be helped by adjuvant radiotherapy after gastrectomy with D2 lymph node dissection. Therefore, to draw any conclusion on the role of adjuvant radiotherapy in gastric adenocarcinoma with D2 gastrectomy, a randomised study is needed.

Initially, our study was designed as a randomised trial, but we failed to achieve this because most patients wanted adjuvant treatment. If we could have completed this trial, we would have been able to draw more precise conclusions about the role of adjuvant treatment. However, given this limitation, our study shows that postoperative chemoradiotherapy may be feasible, that it has an acceptable toxicity and that the locoregional recurrence rate was 16%. To determine the role of adjuvant chemoradiotherapy, we are preparing a matched pair study between an adjuvant chemoradiotherapy group and a surgery alone group. In addition, we plan to undertake a randomised trial to compare adjuvant chemoradiotherapy and chemotherapy alone in patients with gastric adenocarcinoma after D2 gastrectomy.
